# Partial Purification, Characterisation and Gene Detection of Protease by *Lacticaseibacillus paracasei* IN17 from Inasua

**DOI:** 10.21315/tlsr2025.36.2.8

**Published:** 2025-07-31

**Authors:** Naurah Fikriani Zawawi, Nisa Rachmania Mubarik, Laksmi Ambarsari

**Affiliations:** 1 Study Program of Microbiology, Graduate School, IPB University, Jl. Raya Dramaga, Kampus IPB Dramaga, Bogor, 16680 West Java, Indonesia; 2 Department of Biology, Faculty of Mathematics and Natural Sciences, IPB University, Jl. Raya Dramaga, Kampus IPB Dramaga, Bogor, 16680 West Java, Indonesia; 3 Department of Biochemistry, Faculty of Mathematics and Natural Sciences, IPB University, Jl. Raya Dramaga, Kampus IPB Dramaga, Bogor, 16680 West Java, Indonesia

**Keywords:** Ammonium Sulfate, Lactic Acid Bacteria, *prt*P, Skim Milk, Protease

## Abstract

Enzymes are functional proteins that accelerate chemical reactions and reduce the activation energy. *Lacticaseibacillus paracasei*, a lactic acid bacterium, is widely used as a probiotic supplement in the food industry. *L. paracasei* strain IN17 was isolated from Inasua, Indonesian fish fermentation products. It produces an extracellular protease that hydrolyses peptide bonds to produce peptides or amino acids. It is important to characterise bacteria that produce protease to know their activity and potency. This study aims to purify, characterise and detect the protease-encoding gene from *L. paracasei*. Isolates that have been identified were tested qualitatively by measuring the proteolytic index. The optimal production was known for two incubation treatments with static and dynamic conditions. The crude extract was precipitated with ammonium sulfate in the concentration range of 0%–80% (w/v). Enzyme activity characterisation was carried out based on optimum pH and temperature, the effect of cation metal, inhibitor and protease kinetics. The static condition had higher growth and protease-specific activity than the dynamic condition, which were 0.137 U/mg and 0.054 U/mg, respectively, at 18 h of production. Ammonium sulfate saturation at a concentration of 20% (w/v) resulted in a protease purity 7.36. The optimal activity of crude extract and precipitated protease were at pH 7 and 6, respectively. Both crude extract and precipitated protease have an optimal temperature at 30°C, activated by Mn cofactor and inhibited by EDTA with >50% inhibition percentage. This protease represents a metalloprotease. The gene encoding protease (*prt*P) was successfully amplified with ~685 bp visualised in an agarose gel 1% (w/v).

**Highlights**

- Protease produced by *Lacticaseibacillus paracasei* increased under static condition (without shaking) compared to dynamic condition (with shaking).
- Various optimum conditions of protease activity were observed based on pH, temperature, cofactor, inhibitor and enzyme kinetics.
- The *prt*P gene that commonly found in *L. paracasei* from dairy products is also possessed by *L. paracasei* from inasua, fish fermented product.

## INTRODUCTION

Enzymes are functional proteins that accelerate chemical reactions and reduce the activation energy. Enzyme activity in the biochemical processes of living cells occurs by transforming substrates into products (Arsalan & Younus 2018). The demand for enzymes is increasing due to their excellent catalytic efficiency, high specificity and environmental friendliness compared to synthetic catalysts (Tiwari *et al.* 2015). Protease, classified as hydrolases, is the most widely used enzyme, accounting for 60% of the market demand. Protease is an enzyme that plays a role in hydrolysing peptide bonds in proteins to produce peptides or amino acids (Masi *et al.* 2015). Different types of proteases have different properties, depending on catalytic activity, substrate specificity, pH, temperature, active site and stability profile (Naveed *et al.* 2021). These enzymes are involved in protein catabolism, digestion, stress response and cell signalling. The work of proteases is not only limited to biological functions but is also applied in the food and non-food industries. Proteases in the food industry are used to tenderise meat, improve texture, shorten shelf-life, clarify beverages, coagulate milk and produce flavour (Zhang *et al.* 2018). In the non-food industry, proteases are used in detergent production, leather processing, pharmaceuticals, silver recovery and bioremediation (Li *et al.* 2013).

Bacteria, fungi, plants, animals and humans can produce proteases. Microorganisms that produce protease enzymes, especially bacteria, are essential for industrial applications because bacteria can be cultured, produce high yields and could be genetically engineered (Laxman *et al.* 2015). Meanwhile, using plants and animals has limitations due to the influence of climatic conditions and ethical codes. Bacteria that can produce protease enzymes are called proteolytic bacteria. The protease produced by the bacteria is extracellular, allowing it to be secreted directly into the fermentation broth. This extracellular protease breaks down proteins outside the cell, which are then reabsorbed into the cell for metabolic purposes (Ahmad *et al.* 2018). Some bacteria that have been reported to have proteolytic activity include *Acinetobacter*, *Alcaligenes*, *Bacillus*, *Clostridium*,

*Lactobacillus*, *Micrococcus*, *Proteus* and *Pseudomonas* (Kieliszek *et al.* 2021). *Lacticaseibacillus paracasei* are industrial lactic acid bacteria (LAB) widely used for probiotic food products and supplements. These bacteria belong to the phylum Firmicutes, Gram-positive, do not produce endospores and produce lactic acid as the main final product through carbohydrate fermentation (Mora-Villalobos *et al.* 2020). The food and non-food industries utilise LAB because they are generally recognised as safe (GRAS) status, acid resistance, bile tolerance and antimicrobial activity by producing organic acids, H_2_O_2_ and bacteriocins (Costa *et al.* 2019). Several studies reveal this species is generally isolated from dairy food or drink products (Hill *et al.* 2018). In a previous study by Mahulette *et al*. (2018), *L. paracasei* was isolated from Inasua, a fish fermentation product from Central Maluku, Indonesia. It is important to characterise indigenous LAB that produce protease to know their activity and potency. Therefore, purification, characterisation and gene detection of the protease enzyme produced by *L. paracasei* IN17 must be investigated.

## MATERIALS AND METHODS

### Cultivation and Characterisation of IN17 Isolate

Bacterial isolate IN17 was isolated from Inasua Indonesian fish fermentation product, and IPB Culture Collection (IPBCC) was collected on de Mann Rogosa Sharpe Broth (MRSB) medium and then incubated at ±27°C. The isolate strain was grown on de Mann Rogosa Sharpe Agar (MRSA) media to obtain single colonies. The colony was characterised by macroscopic morphology observation, and the cell was characterised by microscopic morphology observation by Gram staining and endospore staining (Madigan *et al.* 2003).

### Identification of IN17 Isolate Based on 16S rRNA Gene

The genome of the IN17 isolate was isolated using the Zymo Research Quick-DNA™ Fungal/Bacterial Miniprep Kit according to the manufacturer’s protocol. The 16S rRNA gene was amplified with polymerase chain reaction (PCR) for 35 cycles using universal primers 63F (5′ CAG GCC TAA CAC ATG CAA GTC 3′) and 1387R (5′ GGG CGG WGT GTA CAA GGC 3′) (Marchesi *et al*. 1998). The temperature and duration of each stage were programmed for denaturation at 95°C for 30 sec, annealing at 55°C for 30 sec, and elongation at 72°C for 1 min. The PCR product was migrated on a 1% (w/v) agarose gel containing Fluorosafe DNA dye at 70 V for 35 min. The amplified genome of IN17 was sequenced by PT. Genetika Science Indonesia (GSI). Nucleotide sequences were processed using the SeqTrace 0.9.0 application and aligned using BLAST-N on the NCBI website (www.ncbi.nlm.nih.gov). A phylogenetic tree was subsequently constructed using the MEGA 11.0.13 programme.

### Measurement of Proteolytic Index

A single colony of IN17 isolate was grown on MRSA with 1% (w/v) skim milk, then incubated at 37°C for 48 h. The presence of a clear zone around the colony indicates positive results. The proteolytic index was calculated according to the following equation.


$$\text{Proteolytic index}=\frac{\text{Clear zone diameter}-\text{colony diameter}}{\text{Colony diameter}}$$

### Determination of Optimum Protease Production

Two loops of IN17 were grown on MRSB and incubated at 37°C for 8 h in static and dynamic conditions. Approximately 1% (v/v) inoculum culture (10^8^ cells/mL) was inoculated into 100 mL MRSB with 1% (w/v) skim milk as production medium and incubated at 37°C with static (without shaking) and dynamic (with shaking) conditions. The culture was collected every 3 h for 24 h, and the optical density was measured at a wavelength of 600 nm. The same cell culture was centrifuged at 15,000 g and 4°C for 10 min. The cell-free supernatant as a crude extract was used for further analysis.

### Protease Specific Activity Assay

#### Protease assay

Enzyme activity was measured using the modified Walter method (1984). Blank, standard and sample were used as test treatments. Approximately 1% (w/v) casein and 0.1 M phosphate buffer were added to each treatment. Distilled water served as the blank, 5 mM tyrosine as the standard and crude extract as the sample. The solutions were incubated at 37°C for 10 min. 0.3 M trichloroacetic acid (TCA) was added to terminate the enzyme activity. Enzyme was included in the blank and standard, while distilled water was added to the sample. The solution was incubated at room temperature for 10 min and then centrifuged at 15,000 g and 4°C for 10 min. The supernatant was reacted with 0.4 M Na_2_CO_3_, followed by the addition of Folin-Ciocalteau reagent diluted with distilled water (1:2). The solutions were incubated for 20 min, and the absorbance was measured at a wavelength of 578 nm. One unit of protease activity is defined as the amount of enzyme capable of producing 1 μmol of tyrosine per minute.

#### Protein content

Protein content was determined using the Bradford method (Bradford 1976), which used bovine serum albumin (BSA) as the standard protein. Approximately 0.05 mL of protease sample was reacted with 2.5 mL of Bradford reagent. The absorbance was measured at 595 nm. The specific activity of the enzyme was calculated according to the following equation.


$$\text{Specific activity}=\frac{\text{Protease activity }(\text{U}/\text{mL})}{\text{Protein content }(\text{mg}/\text{mL})}$$

### Protease Precipitation by Ammonium Sulfate

The crude extract was precipitated with ammonium sulfate in the 0%–80% (w/v) (Scopes 1994). The addition was accompanied by stirring for 1 h at 4°C. The enzyme incubated overnight at 10°C and then centrifuged at 15,000 g for 10 min at 4°C. The pellet was dissolved in 0.1 M phosphate buffer pH 7.0, and the protease activity and protein content were measured.

### Determination of Optimum pH and Temperature on Protease Activity

The optimal pH and temperature were measured to characterise protease activity. The pH range was 4–10, and the temperature range was 20°C–70°C. Protease activity was measured using the modified Walter (1984) method.

### Effect of Metal Cations and Inhibitor on Protease Activity

The effect of metal cations addition on protease activity was tested using divalent cations Ca^2+^, Cu^2+^, Co^2+^, Mg^2+^, Mn^2+^ and Zn^2+^ in chlorine salt under optimum pH and temperature. The effect of protease inhibitor compounds was tested by adding metal chelators and ethylenediaminetetraacetic acid (EDTA). The effect of metal ions and inhibitor compounds was carried out on the concentration of 2 mM and 5 mM enzyme reactions (Afriani *et al*. 2018). Protease activity was measured using the modified Walter (1984) method.

### Determination of K_m_ and V_max_ Values of Protease

The Michaelis constant (K_m_) and maximum velocity of the reaction (V_max_) protease were determined using casein as a substrate at a concentration range of 0.25 mg/mL–20 mg/mL to evaluate the enzyme kinetics. The Lineweaver-Burk curve was created by calculating half of the substrate concentration value against half of the protease activity value. The K_m_ and V_max_ values were calculated using the Michaelis-Menten equation (Sun *et al.* 2019).

### Detection and Analysis of Protease Encoding Gene

The *prt*P/*prt*M protease encoding gene (intergenic region) was amplified with PCR for 30 cycles using specific primers M70F (5′ GCA TGA ATT CAA TGC ACG ATA AAT GAG 3′) and P70R (5′ GCT TGA ATT CGT TGT CGC TGC GGT TGT 3′) (Sfaxi *et al.* 2012). Temperature and duration of each stage were programmed for pre-denaturation at 95°C for 2 min, denaturation at 95°C for 1 min, primer attachment at optimum temperature for 1 min, elongation at 72°C for 1 min and post-extension at 72°C for 5 min. The PCR product was migrated on a 1% (w/v) agarose gel and added Fluorosafe DNA dye at 70 V for 35 min. The sequenced nucleotide was processed using the BioEdit application, translated into amino acids using the Expasy Tool (web.expasy.org/translate/), and aligned with BLAST-P on the NCBI website (https://www.ncbi.nlm.nih.gov/). Amino acid sequence alignments were analysed using Clustal Omega (https://www.ebi.ac.uk/Tools/msa/clustalo), and a phylogenic tree was constructed using the MEGA 11.0.13 programme.

### Data Analysis

The data of the image was analysed using ImageJ for the scale bar. The data of protease activity was obtained from two replicates and analysed using OriginPro 2024b and Microsoft Excel 2019 for standard error.

## RESULTS

### Characterisation and Confirmation of IN17 Based on 16S rRNA

**Figure 1:**
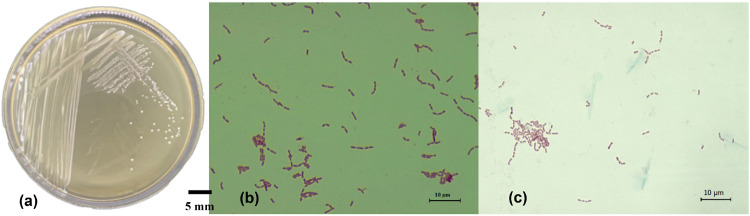
The morphology characterisation of IN17 (a) colonies on MRSA; (b) cells by Gram staining; and (c) endospore staining.

**Figure 2:**
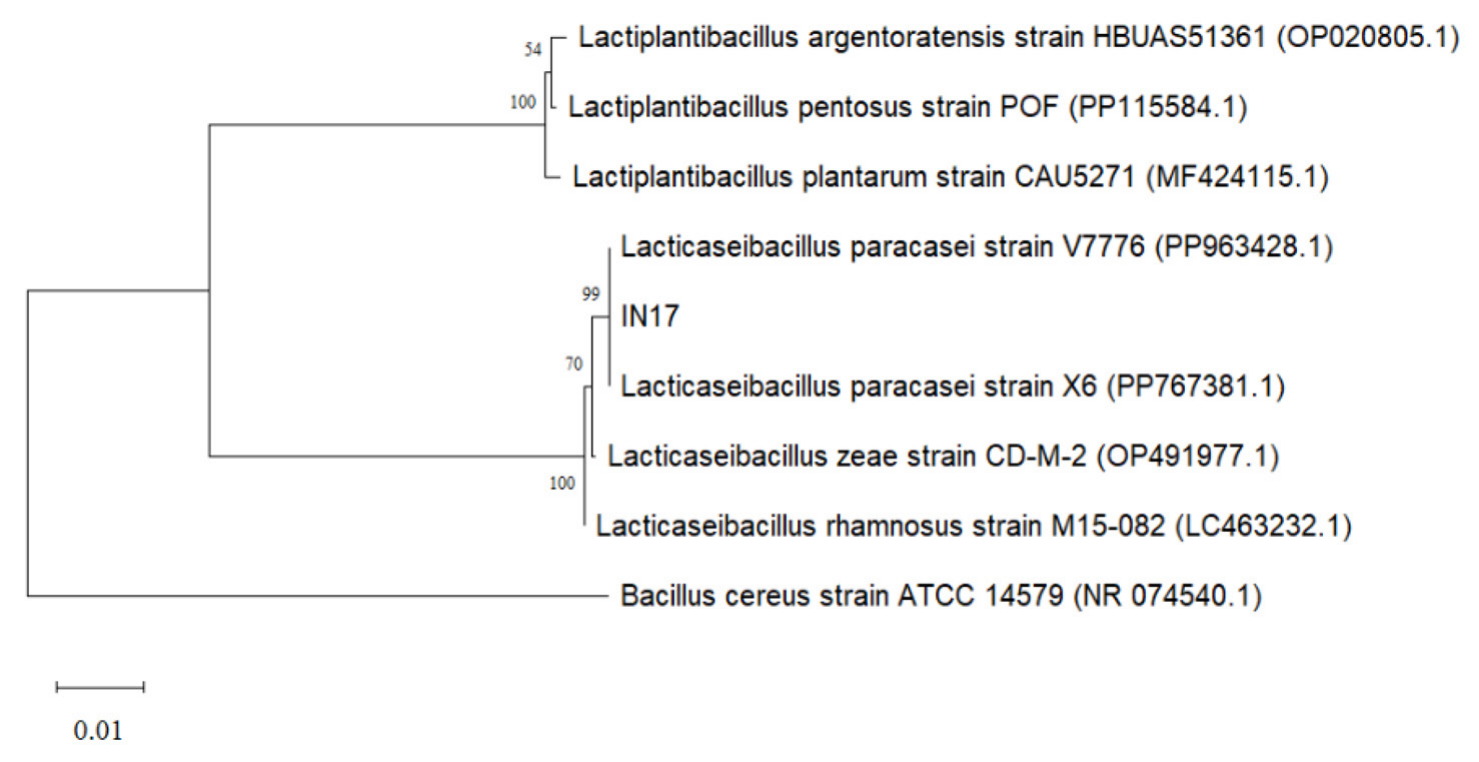
Phylogenetic tree of the relationship between IN17 and comparison isolates using the neighbour-joining method with 1000x bootstrap.

The colonies characteristic of IN17 on MRSA media are milky white, round, flat edges and convex elevations (Fig. 1a). The Gram staining results indicated that the isolate was Gram-positive, as it retained the crystal violet dye, and the cells exhibited a rod shape (Fig. 1b). The isolate did not produce endospores; only bacterial cells were observed without any green endospore structures (Fig. 1c). The 16S rRNA gene identification of the IN17 isolate was conducted to confirm its identity as the isolate obtained from Inasua. The nucleotide sequence homology of IN17 was assessed based on the E-value and identity with reference sequences in the NCBI database. The phylogenetic tree construction (Fig. 2) indicates that IN17 is closely related to *Lacticaseibacillus paracasei* strain X6 and *Lacticaseibacillus paracasei* strain V7776, with a similarity level of 99.92%.

### Bacterial Ability to Produce Extracelullar Protease

**Figure 3:**
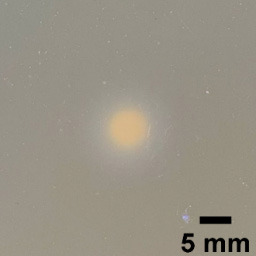
The clear zone formed around the colony of IN17 on MRSA + 1 % (w/v) skim milk.

The formation of a clear zone around the colony indicates an extracellular enzyme. The IN17 isolate exhibits proteolytic activity as it secretes extracellular protease into the media (Fig. 3). This is indicated by the formation of a clear zone with a diameter of 12.3 mm and a colony with a diameter of 5.3 mm, which produces a proteolytic index of 1.3.

### Production of Protease from Cell-Free Supernatant

**Figure 4:**
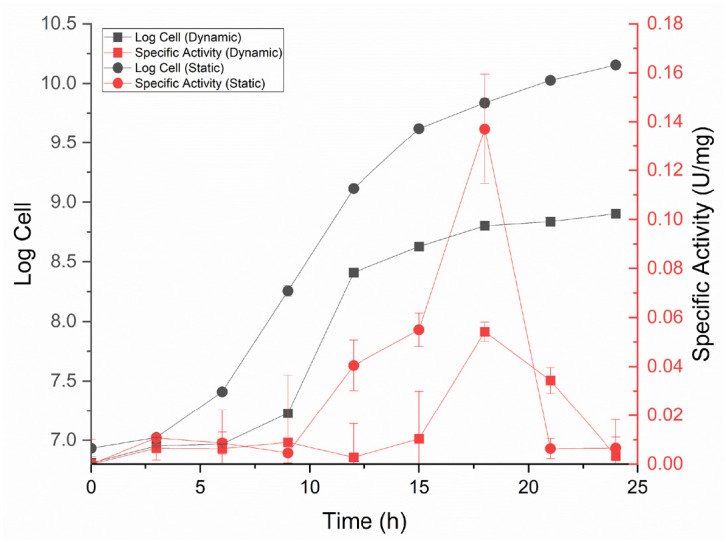
Bacterial cell growth and protease specific activity of IN17 on MRSB + 1% (w/v) skim milk.

Growth and protease production curves were made at 3 h intervals for 24 h. Dynamic and static treatments showed optimum protease production at 18 h at the end of the logarithmic phase. The specific activity of the static treatment was higher than that of the dynamic treatment, with values of 0.137 U/mg and 0.054 U/mg, respectively (Fig. 4).

### Ammonium Sulfate Precipitation

**Figure 5:**
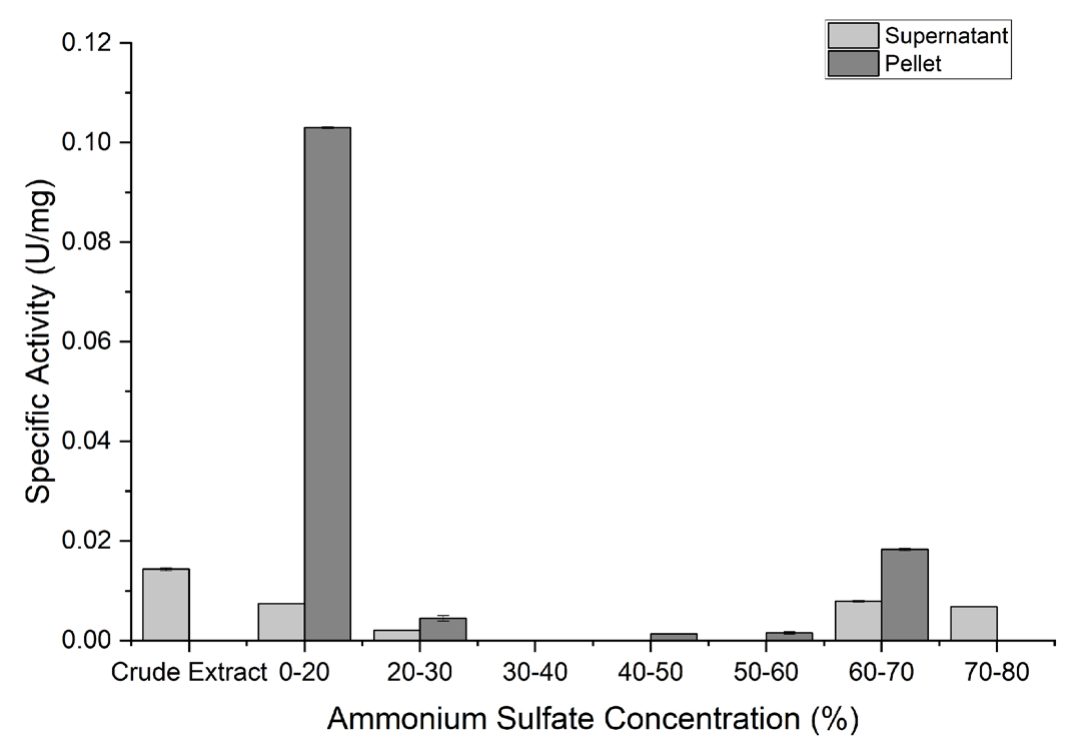
Protease precipitation IN17 by ammonium sulfate at various concentration.

The crude extract of IN17 under optimum conditions was precipitated using ammonium sulfate. This crude extract can be precipitated by ammonium sulfate by increasing protease activity and reducing protein levels. The results of crude protease precipitation with ammonium sulfate at a range of 0%–80% (w/v) saturation indicate that 20% (w/v) ammonium sulfate saturation increases the specific activity of the protease to 0.103 U/mg (Fig. 5). The purity of the precipitated protease increased by 7.36-fold compared to the crude extract, and the protease yield obtained was 14.7% (Table 1).

### The Optimum pH and Temperature of Protease

**Figure 6:**
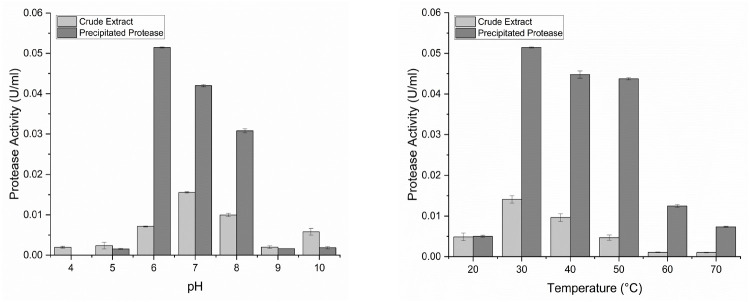
Effect of (a) pH; and (b) temperature on protease activity of crude extract and precipitated protease by IN17.

Characterisation of protease activity is necessary to obtain optimum conditions and enzyme properties in hydrolysing substrates. The static condition had higher specific activity, so enzyme production for characterisation testing was carried out at 18 h statically. The optimum pH and temperature conditions for the protease activity of IN17 were at pH 7 (0.0156 U/mL) and 30°C (0.0141 U/mL). The optimum precipitated protease activity of the IN17 isolate occurred at pH 6 (0.051 U/mL) and 30°C (0.051 U/mL), as shown in Fig. 6.

### The Effect of Cation Metals and Inhibitor

**Figure 7:**
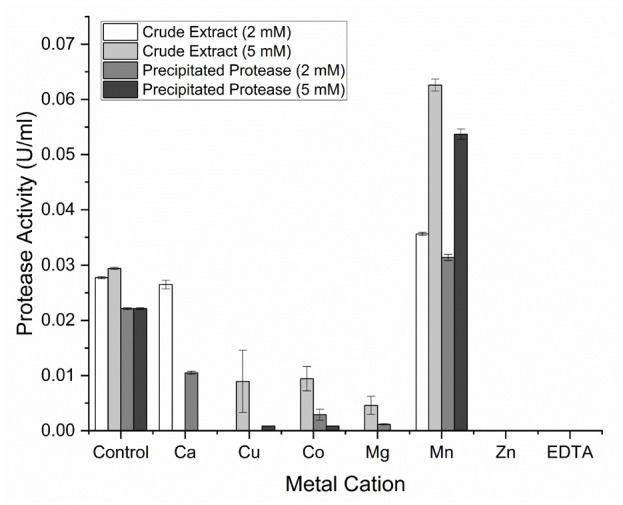
Effect of metal ions and inhibitor on protease activity of crude extract and precipitated protease by IN17.

The administration of Ca^2+^, Cu^2+^, Co^2+^, Mg^2+^ and Zn^2+^ metal ions, as well as the metal chelator EDTA into the enzyme, can reduce the activity of the protease enzyme. Both crude extract and precipitated protease were activated by the Mn^2+^ cofactor at 5 mM, as shown by the increase in protease activity compared to the control. The addition of EDTA at 2 mM and 5 mM inhibited protease activity with a 100% inhibition percentage, as shown in Fig. 7. The crude extract and precipitated protease activity at 5 mM were 0.063 U/mL and 0.054 U/mL, respectively. These results show that the protease of IN17 represents a metalloprotease.

### The K_m_ and V_max_ values

**Figure 8:**
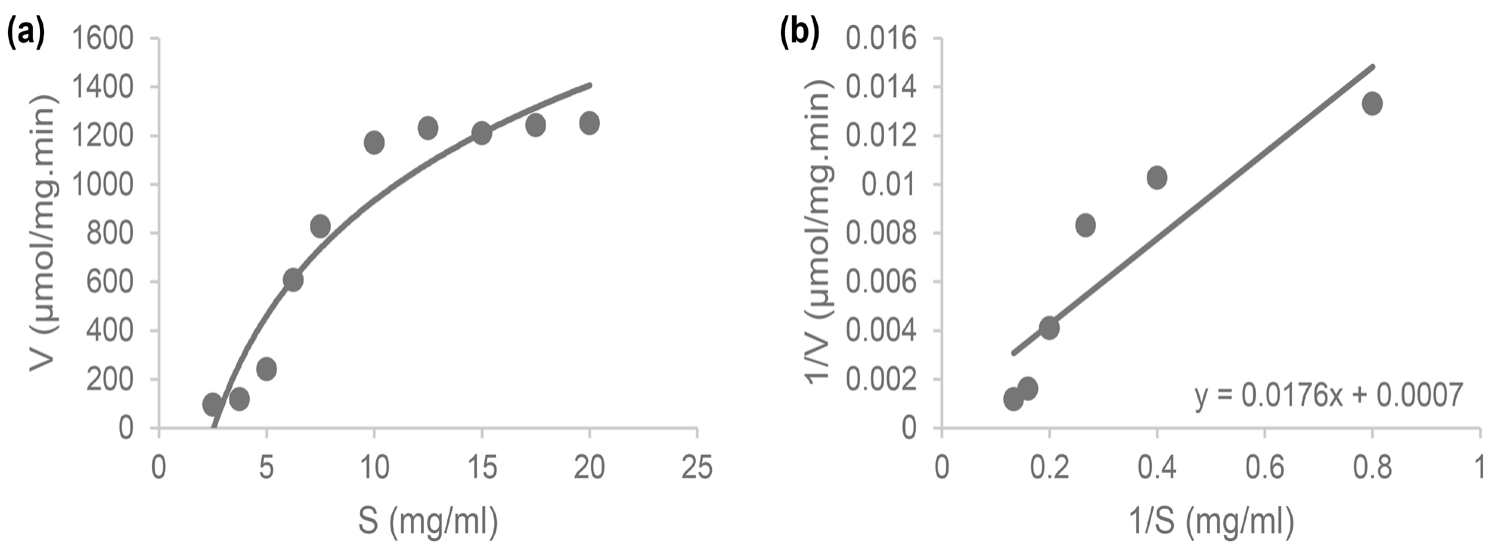
Protease kinetics (a) Michaelis-Menten curve; and (b) Lineweaver-Burk curve.

The K_m_ and V_max_ values are defined by the Michaelis-Menten curve (Fig. 8a) and the Lineweaver-Burk curve (Fig. 8b) for the precipitated protease of IN17. These curves show the Michaelis constant (K_m_) and maximum velocity (V_max_) of the protease. The linear regression equation results in a K_m_ value of 25.14 mg/mL and a V_max_ value of 1428.57 μmol/mg/min.

### Amplification of Protease Encoding Gene (*prt*P/*prt*M)

**Figure 9:**
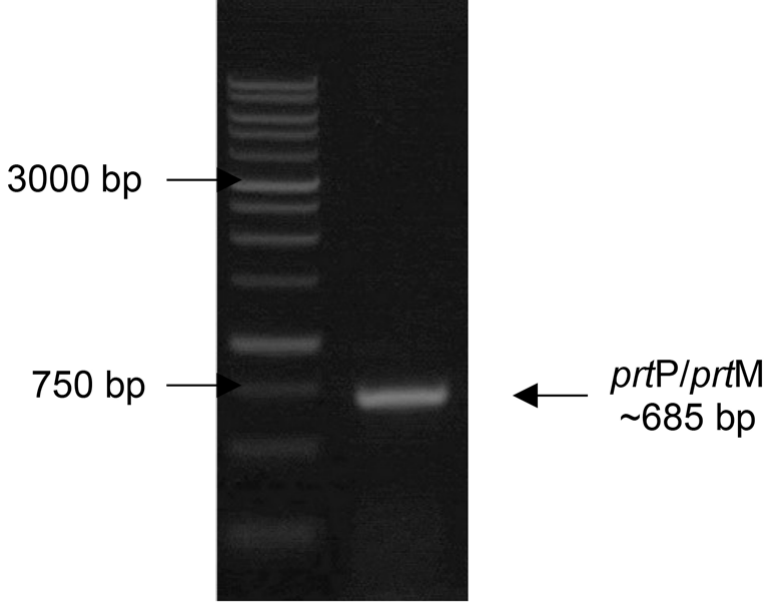
Visualisation of *prt*P/*prt*M gene amplicons (~685) on 1% (w/v) agarose gel. (M) DNA marker 1 kb, (1) IN17.

**Figure 10:**
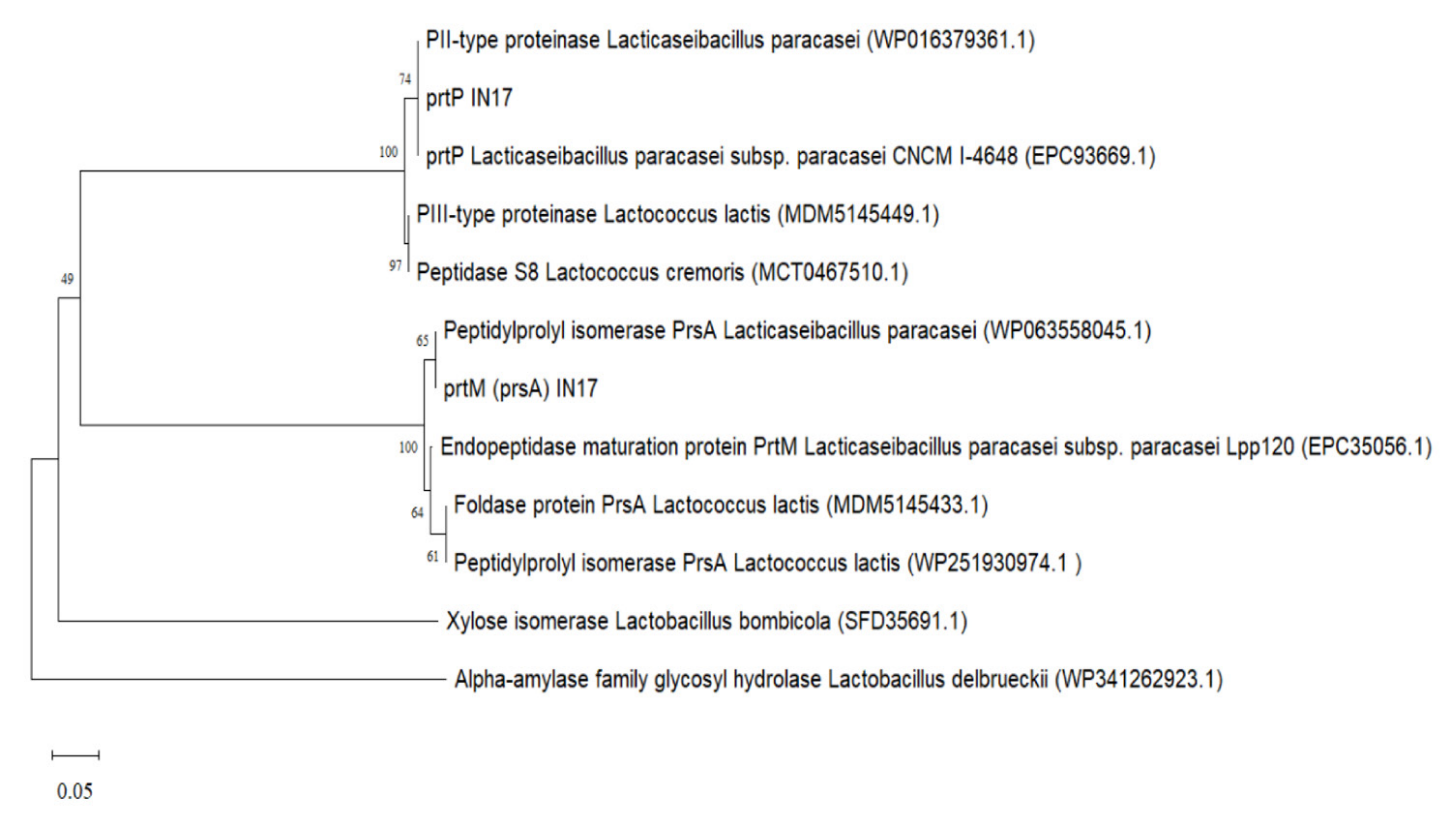
Phenetic tree of the relationship between the *prt*P and *prt*M amino acid sequences of IN17 with reference sequence using the neighbor-joining method with 1,000x bootstrap.

**Figure 11:**
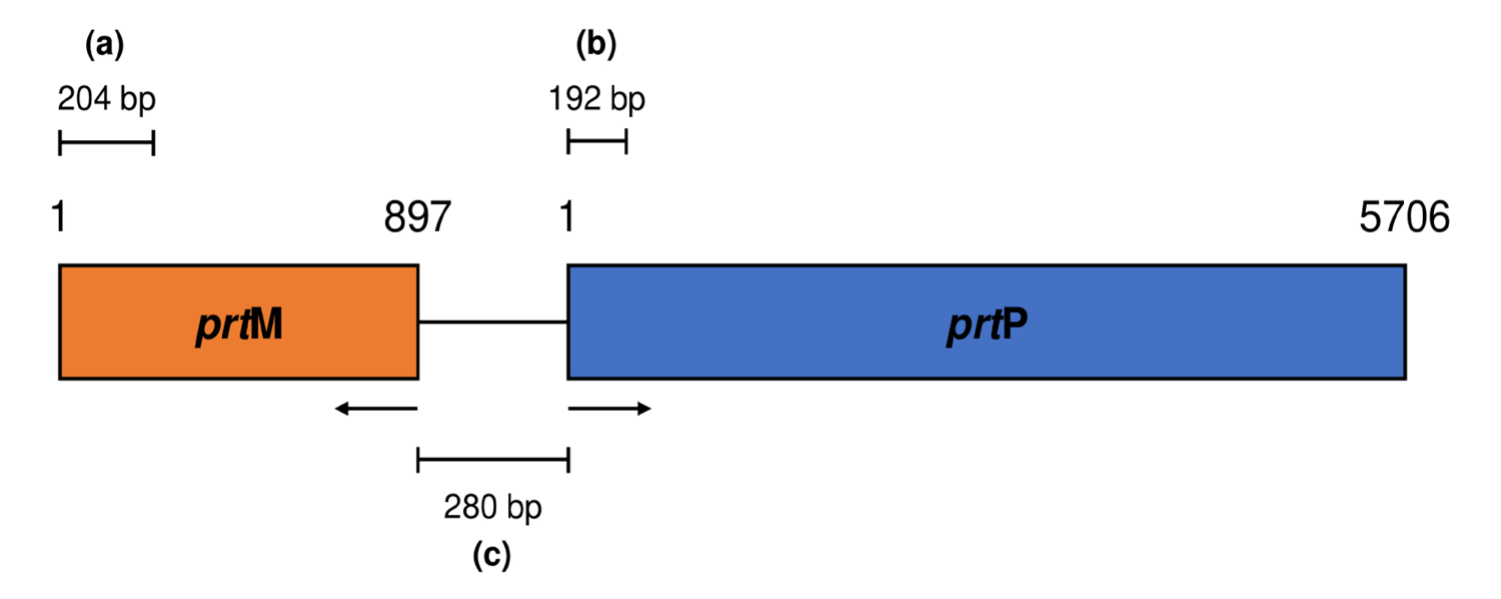
Prediction of *prt*P and *prt*M intergenic region (a) base pairs length of *prt*M, (b) base pairs length of *prt*P, and (c) region of amplified partial sequence of *prt*P/*prt*M in IN17. Arrows show the directions of opposite transcription.

Gene amplification was performed to molecularly detect the presence of protease-encoding genes in the IN17 genome. The PCR product results visualised on the agarose gel showed that the band size obtained was ~685 bp (Fig. 9). The amplicon size indicates that IN17 has the *prt*P gene because it matches the size of the reference amplicon. BLAST-P results on the nucleotide sequence deduced into amino acids showed that two genes were amplified, namely *prt*P and *prt*M (homology: *prs*A). The *prt*P gene is a homolog to P-II type proteinase from *L. paracasei*, while *the prt*M gene is a homolog to peptidylpropyl isomerase from *L. paracasei* (Fig. 10). The amplified *prt*P/*prt*M were predicted to be in the intergenic region with 228 amino acids, as shown in Fig. 11.

## DISCUSSION

The proteolytic isolate in this study is a lactic acid bacterium isolated from fermented marine fish products from Central Maluku called Inasua. This product uses sea fish fermented using salt solution without drying for ±3 months (Nara *et al.* 2013; Mahulette *et al.* 2018). This group of bacteria are Gram-positive and does not produce endospores. The genus *Lactobacillus* comprises 261 species with highly diverse genotypic, phenotypic and ecological characteristics. A study by Zheng *et al.* (2020) evaluated Lactobacillaceae and Leuconostocaceae families based on whole genome sequences. The study proposed reclassifying the genus *Lactobacillus* into *Lactobacillus*, *Paralactobacillus* and 23 new genera, including *Lacticaseibacillus*. The IN17 was previously identified by Mahulette *et al*. (2018) and classified into the *Lactobacillus paracasei* species. Therefore, the name changed from *Lactobacillus paracasei* to *Lacticaseibacillus paracasei*.

Growth and protease enzyme production of IN17 isolate were carried out in two different incubation conditions, which were dynamic and static. The production method was varied to analyse the effect of aeration on cell number and enzyme specific activity. This is based on the growth characteristics of LAB, which are facultative anaerobic, which can grow in aerobic conditions, but the metabolic process becomes anaerobic when there is no O_2_ (Lu & Imlay 2022). The growth of IN17 is higher in static conditions than in dynamic ones. The dynamic condition causes contact between the surface of the media suspension and high oxygen so that the bacteria grow below their optimal growth rate. Meanwhile, static conditions result in much oxygen on the surface of the medium, while bacteria grow at the bottom, so the growth conditions tend to be anaerobic. A study by Rama *et al*. (2019) proved that the number of LAB cells was higher when grown statically than dynamically.

The protease enzyme of IN17 is produced throughout its growth phase. The specific activity of the IN17 isolate enzyme was highest at 18 h, both dynamic and static treatments. However, the specific activity at the optimum time showed higher activity with static condition than with dynamic condition, which amounted to 0.137 U/mg and 0.054 U/mg, respectively. The static condition can increase the specific activity to 2.5-fold higher than the dynamic condition. Based on the growth and production curves, the maximum specific activity occurs at the end of the logarithmic phase. Enzyme production is strongly influenced by bacterial growth. Different protease production conditions resulted in different enzyme activities. Protease is a primary metabolite produced for microbial growth. This primary metabolite is generally produced in the logarithmic phase because bacterial cells are in optimum conditions to metabolise and multiply (Sony 2019).

Crude protease obtained from the highest production of IN17 could be precipitated and increased activity using ammonium sulfate at 20% (w/v). Enzyme precipitation by salt addition is a process of separating proteins from non-proteins. It is caused by the competition between salt ions and proteins in attracting water molecules. Ammonium sulfate is very effective for enzyme precipitation due to its high solubility in water and ability to create an environment of high ionic strength (Gimenes *et al.* 2019). The saturation level of protease precipitation from LAB ranges from 40%–60% (w/v) (Jalkute *et al.* 2017; Zhai *et al*. 2022), which is related to the hydrophilic or hydrophobic character of the amino acids that form the protein of protease. The low saturation of 20% indicates that the protease produced by IN17 has a relatively low solubility compared to other proteins in the crude extract. Enzyme proteins that contain more hydrophobic amino acids require less ammonium sulfate because the protein surface interacts weakly with water, making it easier to disrupt the interaction (Nooralabettu 2014).

The protease activity of IN17 precipitated is optimal at pH 6 and 30°C (0.051 U/mL), while it remains active in the pH range of 6–8 and temperature range of 30°C–50°C. The LAB has a broad pH optimum range for protease activity from acidic to alkaline. Research by Sun *et al.* (2021) showed that *L. fermentum*protease is optimal at pH 6, while Tulini *et al.* (2015) found that *L. paracasei* protease is optimal at pH 6.5. Changes in acidic or alkaline pH conditions can indirectly affect enzyme activity by reducing enzyme activity due to ionisation of the enzyme’s active or passive ionic groups (Afriani *et al.* 2018). The protease crude extracts at pH 4, 5, 9 and 10 have higher activity than the precipitation results possibly due to proteases that tend to be more stable in natural environments, which contain various protective compounds. After precipitation, the protease may be more susceptible to pH conditions that are too acidic or too basic. This means that the precipitated protease has characteristics that are unable to maintain its protein structure in highly acidic or highly alkaline conditions. The typical optimal temperature range for protease activity is generally between 30°C–50°C. Sun et al. (2021) found the optimum temperature for *L. brevis* protease to be 35°C, while Tulini *et al.* (2015) found the optimum temperature for *L. paracasei* to be 42°C. Enzyme stability depends on hydrogen bonding, hydrophobic interactions, ionic interactions, and disulfide bonds. Enzyme performance is maintained when amino acids form protein conformations resistant to denaturation effects (Afriani *et al.* 2018).

Heavy metal ions significantly influence enzyme activity as nonprotein components known as cofactors. These ions can act as inhibitors and activators. The addition of Ca^2+^, Cu^2+^, Co^2+^, Mg^2+^ and Zn^2+^ ions decreases protease activity, hence termed enzyme inhibitors. The crude extract and precipitated protease activity increased 41.64% and 142.53%, respectively, upon adding 2 mM and 5 mM Mn^2+^. The role of Mn^2+^ in protease function is confirmed by adding a metal chelator compound EDTA, which results in a 100% decrease in protease activity. This condition exemplifies metalloproteases, enzymes whose activity depends on the presence of divalent metal ions at their catalytic sites, mainly in the presences of Mg^2+^, Mn^2+^, Ca^2+^ and Zn^2+^ (Sun *et al*. 2021; Gimenes *et al.* 2019). The Mn^2+^ ions are uncommon cofactors whose presence may be due to architecture of the active site and environmental conditions. The addition of EDTA will bind ions and cofactors to enzymes that play a role in maintaining the stability of the enzyme. These findings are consistent with those of Tulini *et al*. (2015), who successfully identified a metalloprotease from *L. paracasei* in their study.

Enzyme kinetics (V_max_ and K_m_ values) are important to know as specific values of protease ability. Even though the same protease enzyme, each protease has different V_max_ (maximum velocity) and K_m_ (Michaelis constant) values. The kinetic parameters of IN17 protease were studied using the precipitated protease at the optimum conditions of 30°C and pH 6. The protease produced by IN17 requires 25.14 mg/mL of casein substrate to achieve half of its maximum reaction velocity. A more minor K_m_ relative to a larger V_max_ indicates the enzyme is effective in low substrate conditions, whereas a larger K_m_ relative to a smaller V_max_ suggests effectiveness in high substrate concentrations. In this study, the K_m_ value of protease IN17 is lower compared to Sulthoniyah *et al.* (2015), which is at 79.7 mg/mL, but higher compared to Sun *et al*. (2019) at 17.1 mg/mL.

Successful amplification of the partial *prt*P/*prt*M gene was indicated by an amplicon size of ~685 bp in agreement with Sfaxi *et al.* (2012) who used *L. paracasei* isolated from tarag, a typical Mongolian fermented milk food. This study shows that the *prt*P gene, which is commonly found in *L. paracasei* from dairy products, is also possessed by *L. paracasei* from inasua, a typical Indonesian fermented fish products. The gene *prt*P encodes the cell envelope protease (CEP) and is highly expressed in the probiotic strain *L. paracasei* BL312 (VSL#3) (Coll-Marqués *et al.* 2020). Previous studies have confirmed that proteolytic *L. paracasei* strains carry both *prt*P and *prt*M (*prs*A) genes, where *prt*M is responsible for the maturation of *prt*P. These genes are located adjacently on the chromosome and are transcribed in opposite directions, with their promoters situated in the intergenic region (Alcántara *et al.* 2016). *L. paracasei* possesses the *prt*P and *prt*M genes, but *prt*M has not been identified in *Lactobacillus helveticus, Lactobacillus delbrueckii* subsp. *bulgaricus*, and *Lactobacillus thermophilus*in the vicinity of the protease gene cluster (Brown *et al*. 2017; Kieliszek *et al*. 2021).

## CONCLUSION

Production of protease enzyme by *Lactobacillus paracasei* IN17 was optimal at 18 h of static incubation and could be precipitated by adding ammonium sulfate. The increase in protease activity of the concentrated results occurred at 20% ammonium sulfate concentration with a purity value of 7.36. The character of protease produced by *L. paracasei* has optimum activity at pH 6, temperature 30°C, activated by Mn cofactor and 100% inhibited by EDTA, so it is a metalloprotease. A total of 25.14 mg/mL of casein substrate was required to achieve half of the maximum protease reaction speed of 1428.57 μmol/mg/min. The gene encoding protease (partial *prt*P/*prt*M) was successfully amplified with an amplicons size of ~685 bp.

## Figures and Tables

**Table 1:** Purification steps of protease activity by IN17.

Purification step	Volume (mL)	Total activity (U)	Total protein (mg)	Specific activity (U/mg)	Fold	Yield (%)
Crude extract	50	0.15	11.4	0.014	1	100
Ammonium sulfate 20% (w/v)	1	0.022	0.214	0.103	7.36	14.7
